# Enhancing thermal tolerance of *Aspergillus niger* PhyA phytase directed by structural comparison and computational simulation

**DOI:** 10.1186/s12896-018-0445-y

**Published:** 2018-06-01

**Authors:** Nanyu Han, Huabiao Miao, Tingting Yu, Bo Xu, Yunjuan Yang, Qian Wu, Rui Zhang, Zunxi Huang

**Affiliations:** 1 School of Life Sciences, Yunnan Normal University, Kunming, 650500 China; 2 Key Laboratory of Yunnan for Biomass Energy and Biotechnology of Environment, Yunnan Normal University, Kunming, 650500 China; 3 Engineering Research Center of Sustainable and Utilization of Biomass Energy, Ministry of Education, Kunming, 650500 China

**Keywords:** Phytase, Thermostability, Site-directed mutagenesis, Homologous structural comparison, Molecular dynamics simulation

## Abstract

**Background:**

Phytase supplied in feeds for monogastric animals is important for improving nutrient uptake and reducing phosphorous pollution. High-thermostability phytases are particularly desirable due to their ability to withstand transient high temperatures during feed pelleting procedures. A comparison of crystal structures of the widely used industrial *Aspergillus niger* PhyA phytase (AnP) with its close homolog, the thermostable *Aspergillus fumigatus* phytase (AfP), suggests 18 residues in three segments associated with thermostability. In this work, we aim to improve the thermostability of AnP through site-directed mutagenesis. We identified favorable mutations based on structural comparison of homologous phytases and molecular dynamics simulations.

**Results:**

A recombinant phytase (AnP-M1) was created by substituting 18 residues in AnP with their AfP analogs. AnP-M1 exhibited greater thermostability than AnP at 70 °C. Molecular dynamics simulations suggested newly formed hydrogen bonding interactions with nine substituted residues give rise to the improved themostability. Thus, another recombinant phytase (AnP-M2) with just these nine point substitutions was created. AnP-M2 demonstrated superior thermostability among all AnPs at ≥70 °C: AnP-M2 maintained 56% of the maximal activity after incubation at 80 °C for 1 h; AnP-M2 retained 30-percentage points greater residual activity than that of AnP and AnP-M1 after 1 h incubation at 90 °C.

**Conclusions:**

The resulting AnP-M2 is an attractive candidate in industrial applications, and the nine substitutions in AnP-M2 are advantageous for phytase thermostability. This work demonstrates that a strategy combining structural comparison of homologous enzymes and computational simulation to focus on important interactions is an effective method for obtaining a thermostable enzyme.

**Electronic supplementary material:**

The online version of this article (10.1186/s12896-018-0445-y) contains supplementary material, which is available to authorized users.

## Background

Phytate (*myo*-inositol hexakisphosphate) is the primary form of stored phosphorus in most plants and consequently, in animal feeds [1]. Due to inadequate levels of digestive enzymes in the gastrointestinal tracts of monogastric animals (swine, poultry, fish, etc.), phytate cannot be utilized directly [2]. Supplemental inorganic phosphate can partially compensate for the deficiency, but excessive phytate phosphorus in animal excretions induces severe ecological problems [3, 4]. The digestive enzyme phytase can hydrolyze phytic acid or phytate into lower inositol phosphates [5]. Thus, phytases supplied in animal feeds not only improve phytate utilization in monogastric animals, but also alleviate phytate phosphorus pollution [6].

The first commercial phytase products were introduced 20 years ago [7]. Currently, over 60% of feed products include phytase [8]. To lower production costs, novel thermostable phytases capable of withstanding the transient high temperatures (80–85 °C) of the feed production pelleting process are in high demand [9].

The *Aspergillus niger* PhyA phytase (AnP) exhibits superior enzymatic activity compared to other fungal phytases [5]. Specifically, AnP exhibited considerably higher specific activity (102.5 U/mg) than the *Aspergillus fumigatus* phytase (AfP) (26.5 U/mg) [10]. Moreover, AnP is an acidic phosphatase that optimally active in the pH range prevalent in the digestive tract [2, 10]. However, when exposed to pelleting process conditions, AnP loses 70–80% of its enzymatic activity [11]. In contrast, the phytase from AfP, which shares 66% sequence identity and has similar overall crystal structure [12], is well known for its heat resistance: AfP retains 90% of its initial enzymatic activity after heating to 100 °C for 20 min [13]. Thus, AfP may act as a guide for AnP enzymatic engineering for both high activity and high thermostability [14].

Based on the above hypothesis, Tao Xiang and coworkers proposed three segments that could contribute to the thermostability of AfP from comparing crystal structures of AnP and AfP [12]. We confirmed these sites and constructed a recombinant phytase (AnP-M1) by substituting residues in the three segments in AnP with residues at corresponding sites in AfP. As expected, the resultant recombinant demonstrated higher thermostability than the parent AnP. We explored potential AnP-M1 heat resistant mechanisms using molecular dynamics (MD) simulations, and nine of the 18 mutated residues in AnP-M1 exhibited potential interactions that could contribute to this. Subsequently, we created our second recombinant phytase (AnP-M2) by substituting just the nine proposed thermostability-enhancing residues into AnP. This new variant (AnP-M2) demonstrated even greater thermal tolerance.

This work combines structural comparison and computational simulation to guide the design of a thermostable phytase. The most promising new variant has particular potential for the animal feed industry.

## Results

### AnP-M1 showed improved thermostability

**Fig. 1:**
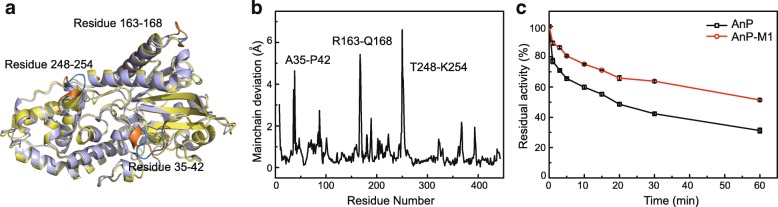
Structural comparison of homologous phytases and thermostability assays of the first recombinant. Structural comparison (**a**) and mainchain deviation (**b**) between AnP (tv_blue) and AfP (tv_yellow). Residual activity of AnP (black) and AnP-M1 (red) incubated at 70 °C for 1 h (**c**)

Inspired by the work done by Tao Xiang and coworkers [12], mainchain deviations were re-calculated. After superimposing AnP and AfP crystal structures, Cα atoms between analogous residues in three segments (A35-P42, R163-Q168, T248-K254 in AnP) were shown to have distances greater than 3 Å (Fig. 1a, b). This result is consistent with the findings from Tao Xiang et al.

The first recombinant phytase (AnP-M1) was created by substituting residues from AnP in the above three segments with the corresponding residues in AfP by site-directed mutagenesis (35-ANESVISP-42, 163-RAQPGQ-168, and 248-TSTVDTK-254 in AnP correspond to 35-EDELSVSS-42, 163-GATNRA-168, and 248-RTSDASQ-254 in AfP). Thermostability assays revealed that AnP-M1 retained greater activity after treatment at 70 °C for 1 h compared to AnP (Fig. 1c). AnP-M1 retained 51% activity while AnP retained 31% activity.

### Hydrogen bonds in AnP-M1 govern the improved thermostability

Generally, creating an enzyme with improved thermostability usually requires combining multiple amino acid exchanges, each of which slightly increases the unfolding temperature of the protein. Some example mechanisms are: new hydrogen bonds, new disulfide bridges, β-turns or flexible termini stabilization, hydrophobic packing enhancement, or α-helix or β-sheet stabilization [15]. One study showed that the hydrogen bonding appears to be the most important factor for thermostability based on analysis of enzymes from 16 families [16]. Specifically, increased hydrogen bonding interactions are observed in the three segments of AfP based on structural analysis [12]. To identify the contribution of particular residues towards improving thermostability, we performed MD simulations of AnP and AnP-M1 at two temperatures (50 °C and 70 °C). Both AnP and AnP-M1 were stable during the 50 ns simulations at two temperatures, with the overall root-mean-square deviations (RMSD) of the heavy atoms with respect to the initial structure less than 0.35 nm (Additional file 1: Figure S1). After investigating the hydrogen bond patterns, we identified nine mutated residues in AnP-M1 that form new hydrogen bonding interactions that could explain the improved thermostability.

**Fig. 2:**
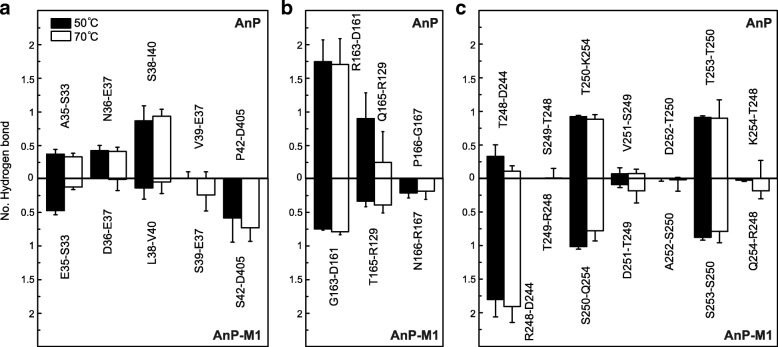
Statistics of hydrogen bonding with mutated residues. Statistics of hydrogen bonds connecting residues in segment 1–3 (**a-c**, respectively) and nearby residues for AnP and AnP-M1 from simulations at 50 °C and 70 °C

Hydrogen bonds connecting segment-1 residues were monitored throughout the entire simulation in each phytase. Most hydrogen bonds connecting 35-ANESVISP-42 and nearby residues in AnP persisted at both temperatures (Fig. 2a). The hydrogen bond connecting AnP A35 and S33 was well preserved at both temperatures (36% at 50 °C and 33% at 70 °C). The probability of hydrogen bonding between AnP N36 and E37 is 42% at 50 °C and 40% at 70 °C. The probability of hydrogen bonding between S38 and I40 in AnP increased to 93% at 70 °C. In AnP-M1, the formation probabilities of three hydrogen bond pairs, E35-S33, D36-E37, and L38-V40, were less than those in AnP.

Interestingly, the probability of hydrogen bonding between S39 and E37 in AnP-M1 increased to 25% at 70 °C. Additionally, another hydrogen bond connecting AnP-M1 S42 and D405 was well preserved at both temperatures: probabilities of hydrogen bonding between S42 and D405 were 59 and 74% at 50 °C and 70 °C, respectively. However, hydrogen bonding pairs V39-E37 and P42-D405 in AnP were rarely observed. In summary, two newly formed hydrogen bond pairs, S39-E37 and S42-D405, may contribute to the enhanced thermostability for AnP-M1, and the mutated serines at position 39 and 42 in segment-1 are critical for the interactions.

Hydrogen bond interactions within segment-2 residues in AnP and AnP-M1 were also analyzed (Fig. 2b). AnP R163 and D161 formed 1.74 and 1.70 hydrogen bonds at 50 °C and 70 °C, respectively. In contrast, AnP-M1 G163 and D161 formed no more than 0.8 hydrogen bonds at both temperatures. In AnP, the probability of a hydrogen bond connecting Q165 and R129 was 89% at 50 °C, decreasing to 24% at 70 °C, indicating a flexible interaction between Q165 and R129 in AnP at elevated temperature. In contrast, the hydrogen bond between T165 and R129 in AnP-M1 was well maintained and increased to 40% at 70 °C, suggesting a stable and favorable contact at higher temperature. Likewise, the probability of hydrogen bonding between AnP-M1 N166 and R167 was approximately 20% at both temperatures, while no hydrogen bond was observed between AnP P166 and G167 at either temperature. In summary, AnP-M1 segment-2 hydrogen bond pairs R129-T165 and N166-R167 may contribute to the enhanced thermostability.

Hydrogen bonds formed within segment-3 residues in AnP (248-TSTVDTK-254) and AnP-M1 (248-RTSDASQ-254) were also compared (Fig. 2c). It is evident that R248 and D244 in AnP-M1 formed almost 2 hydrogen bonds at both temperatures, and the probability of this interaction is 4-fold greater than that between the corresponding AnP T248 and D244. Almost no contact was monitored between residues 248 and 249 in either AnP or AnP-M1. Although AnP-M1 S250 and Q254 formed 1.01 and 0.78 hydrogen bonds at the two temperatures, AnP T250 and K254 formed more hydrogen bonds at 70 °C (0.88), indicating a more stable interaction between AnP T250 and K254 at 70 °C. Similarly, the hydrogen bond between AnP-M1 S253 and S250 is not as strong as that between AnP T253 and T250. In contrast, D251, A252, and Q254 in AnP-M1 were associated with higher probability in forming hydrogen bonds with nearby residues than those in AnP. In summary, AnP-M1 segment-3’s hydrogen bonds within R248, D251, A252, and Q254 may contribute to the improved thermostability.

**Fig. 3:**
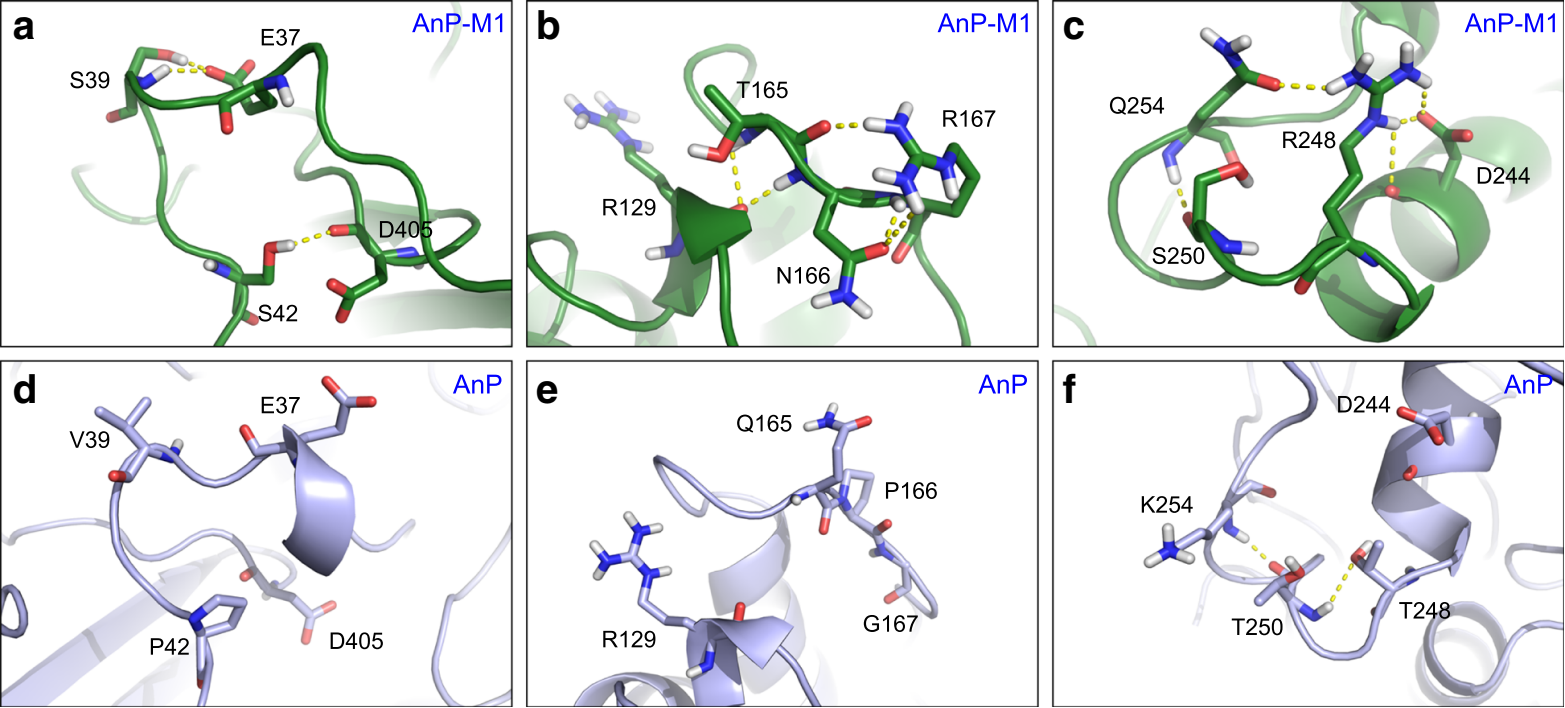
Comparison of hydrogen bonding networks formed by mutated residues in AnP and AnP-M1. Illustration of the newly formed hydrogen bonding network in AnP-M1 (**a**-**c**) and analogous locations in AnP (**d**-**f**). Representative structures are cluster centers from clustering analysis

In light of the above, not all the mutagenesis sites demonstrated stable or increased hydrogen bonding interactions with nearby residues in AnP-M1, suggesting that only a subset of the 18 point mutations contributed to the improved thermostability. Comparing number of hydrogen bonds formed with the substituted residues in AnP and AnP-M1, we concluded that S39 and S42 in segment-1 (Fig. 3a, d); T165, N166, and R167 in segment-2 (Fig. 3b, e); and R248, D251, A252, and Q254 in segment-3 (Fig. 3c, f) make the greatest contribution to the enhanced AnP-M1 thermostability.

### AnP-M2 showed superior thermal tolerance among all AnPs

To further increase AnP thermostability, we performed another round of site-directed mutagenesis based on lessons learned from our analysis of AnP-M1. In total, nine AnP residues were substituted to AfP residues at the equivalent sites (S39 V, S42P, T165Q, N166P, R167G, R248T, D251V, A252D, and Q254K). This nonuple mutant is denoted AnP-M2.

**Fig. 4:**
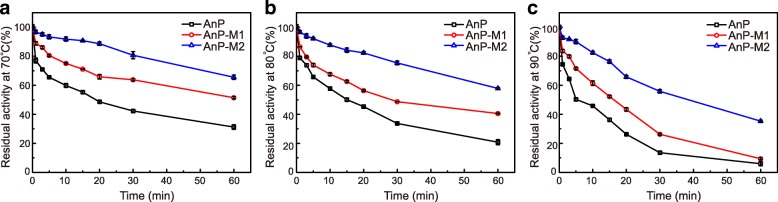
Thermostability assays of all three AnPs. Residual activities of AnP (black), AnP-M1 (red), and AnP-M2 (blue) incubated at 70 °C (**a**), 80 °C (**b**), and 90 °C (**c**) for 1 h

To evaluate thermostability, we measured the residual activities of AnP, AnP-M1 and AnP-M2 after 1 h incubation at three temperatures (70, 80, and 90 °C). The two mutants showed promising improvements in residual activity, and the nonuple mutant AnP-M2 exhibited the highest thermal tolerance. Specifically, AnP-M2 retained 66% activity after 1 h incubation at 70 °C, AnP-M1 retained 51% at the same condition, and AnP retained markedly less (31%) (Fig. 4a). After 1 h incubation at 80 °C, AnP-M2, AnP-M1 and AnP retained 58, 41 and 21% activity, respectively (Fig. 4b). After incubation at 90 °C for 1 h, AnP-M2 retained 30-percentage points greater residual activity than that of the AnP and AnP-M1 (Fig. 4c). These results highlight that the nine substitutions in AnP-M2 are advantageous for phytase thermostability.

### Kinetic analysis of AnPs

Kinetic analysis showed apparent *K*_*m*_ values for AnP, AnP-M1 and AnP-M2 of 289.8, 116.5 and 135.2 μM, respectively (Table 1). The smaller Michaelis constant (*K*_*m*_) of both mutants indicate an increase in kinetic efficiency compared to that of AnP. Comparing *k*_*cat*_*/K*_*m*_ values of three AnPs, AnP-M1 has the highest catalytic efficiency, followed by AnP and the AnP-M2. Kinetic analysis suggests that the substitutions in AnP-M1 and AnP-M2, which we designed primarily to improve thermostability, enhanced substrate binding affinity (*K*_*m*_) by two-fold; however, the *k*_*cat*_ values of both mutants were decreased to the same extent and *k*_*cat*_*/K*_*m*_ which represents catalytic efficiency was slightly decreased for AnP-M2 versus AnP-M1.

**Table 1:** Kinetics of AnP, AnP-M1 and AnP-M2

Enzymes	*K*_*m*_ (μM)	*V*_*max*_ (μmol/min/mg)	*k*_*cat*_ (/s)	*k*_*cat*_/*K*_*m*_ (/s/μM)
AnP	289.8 ± 13.8	5954.2 ± 63.8	4885.4 ± 52.1	16.9 ± 1.7
AnP-M1	116.5 ± 9.2	3117.1 ± 55.1	2557.6 ± 44.9	22.0 ± 1.8
AnP-M2	135.2 ± 8.8	2484.4 ± 59.1	2042.6 ± 48.2	15.1 ± 1.8

## Discussion

In order to create AnP variants with substantially increased thermostability, we performed two rounds of rationally designed site-directed mutagenesis. A crystal structure comparison between regular AnP and a particularly thermostable homolog, AfP, indicated three segments that may influence thermostability. MD simulations on a mutant AnP created based these comparisons, AnP-M1, explored heat resistance mechanisms and pointed out nine particular thermostability-enhancing residues. We created a final recombinant, AnP-M2, with superior thermostability, enhanced substrate binding affinity, and decreased catalytic efficiency compared to other AnPs.

A previous study on phytase thermostability focused on three residues derived from structural modeling, indicated that hydrogen bond network and ionic interaction formed with the three residues support the high thermostability of AfP [17]. These three residues are present in both our full 18-residue homology-based mutant and refined 9-residue MD-based mutant. Similarly, our study discovered that hydrogen bonding interactions formed with the nine substituted residues account for the improved thermostability.

The fungal histidine acid phytases (HAPs) possess an extra N-terminal region before the first β-strand of the α/β domain. This region folds into short helices and loops. Structural studies suggest that the N-terminal region in tetrameric phytase extend outward and form part of the interface of the dimer and tetramer structures [18]. Residues 35–42 in AnP segment-1 are located within the fungal HAP’s unique N-terminal region. Therefore, the newly formed hydrogen bond pairs S39-E37 and S42-D405 in segment-1 may play a pivotal role in enhancing the thermal tolerance of phytase mutants through stabilizing the local structure in the N-terminal region.

**Fig. 5:**
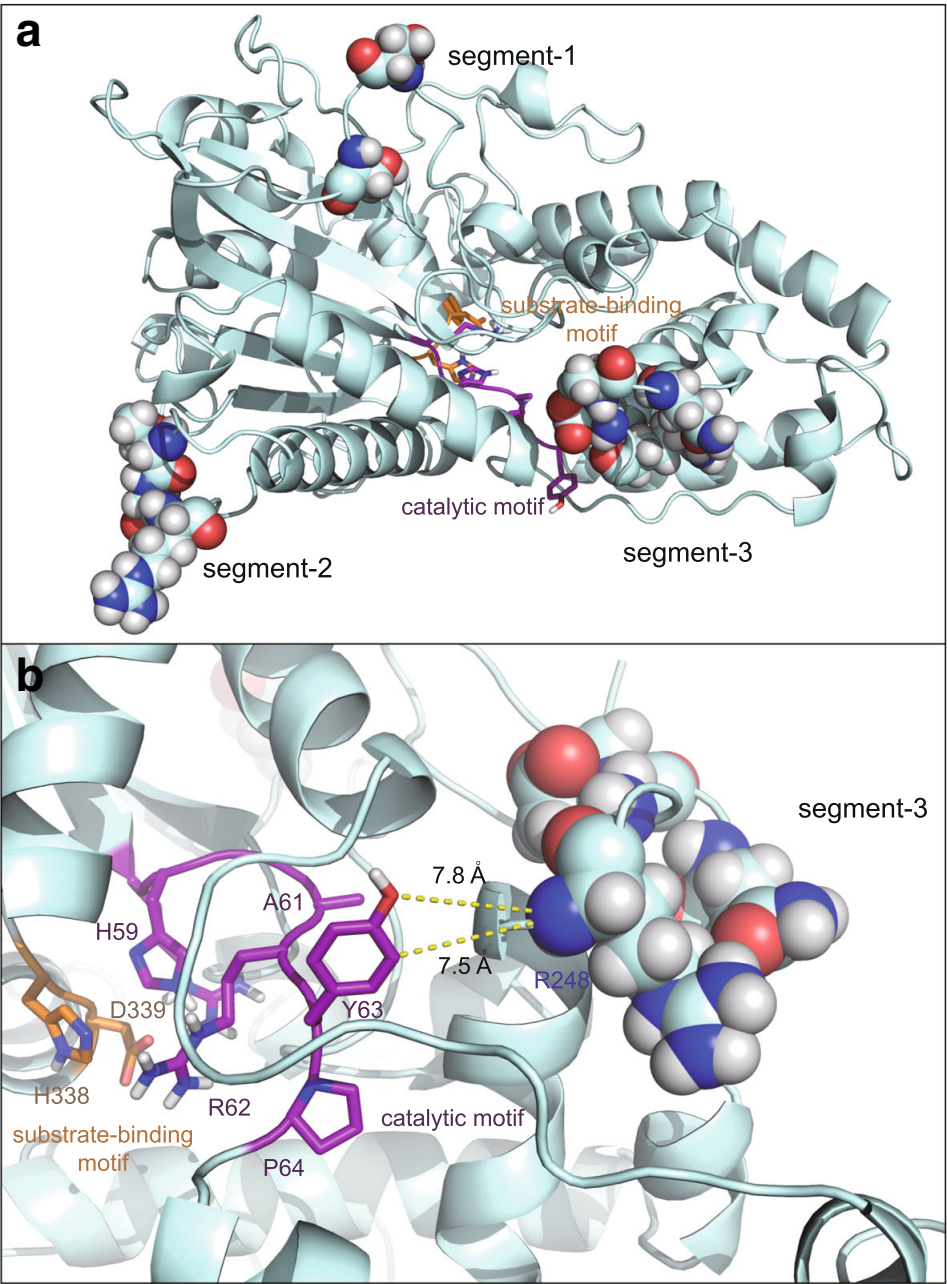
Illustration of mutated and catalytic residues in AnP-M2. Mutated residues in AnP-M2 are shown in spheres, substrate binding motif (orange) and catalytic motif (purple) are shown in cartoon (**a**). Distances between R248 in segment-3 of AnP-M2 and Y63 of the catalytic motif were shown in yellow dash (**b**)

Although AnP-M2 has the highest thermostability, it has slightly decreased catalytic efficiency compared to that of AnP. The consensus catalytic motif 58-RHGARYP-64 and substrate-binding motif 338-HD-339 are located in the deep substrate-binding cleft at the interface of the large α/β domain and the small α domain (Fig. 5a). Structural analysis shows that mutated sites from segment-3, located at the small α domain, are close to the catalytic and substrate-binding motifs, with a ~ 8 Å distance between the closest segment-3 and substrate-binding residues (Fig. 5b). One previous effort to improve enzyme thermostability only mutated residues > 10 Å away from the active site in order to avoid such possible interference [19]. Mutations in the AnP-M2 segment-3 may affect local movement, thus influencing catalysis. This conjecture requires experimental studies which could consist of single point mutations of AnP-M2 segment-3 residues to identify catalytic efficiency influencing residue (s). This provides a next step for designing a new variant with both high thermostability and high catalytic efficiency.

## Conclusions

Our work demonstrates the improvement of an enzyme using a multipart rational design strategy: identifying residues from homologs with desirable properties followed by determining important mechanisms from MD simulations and then final design refinement. The final designed *A.niger* PhyA variant also provides new insight into the design of thermostable phytases. Additional enzyme properties could also be modified using this combined method.

## Methods

### Materials

High fidelity DNA polymerase, restriction endonuclease (*Eco*RI, *Not*I) and dNTP from TaKaRa (Otsu, Japan). Plasmid mini-prep kit and DNA gel extraction kit from Omega (Taipei, USA). One-step cloning kit from Vazyme biotech (Nanjing, China). Fast MultiSite Mutagenesis System, Bradford protein assay kit, *Escherichia coli* Trans I-T1 cells and *E.coli* DMT cells from TransGen (Beijing, China). All other chemicals were of analytical grade and commercially available.

### Gene cloning and site-directed mutagenesis

The *AnP* sequence came from the *Aspergill*u*s niger* PhyA gene (GenBank: Z16414) in the pMD19-T vector [20]. Genes of mutants (*AnP-M1* and *AnP-M2*) were constructed by introducing mutations to *AnP* through site-directed mutagenesis using the Fast MultiSite System according to the manufacturer’s instructions. PCR cycling conditions consisted of an initial step of 5 min at 94 °C, followed by 30 cycles of 30 s at 94 °C, 1 min at 55 °C, and 3 min 30 s at 72 °C. Forward and reverse primers for *AnP-M1* and *AnP-M2* are listed in Additional file 2: Table S1. The *AnP-M1* and *AnP-M2* PCR products in the pPIC9K vector were confirmed by DNA sequencing.

### Protein expression and purification

*P. pastoris* GS115 competent cells were transformed with the plasmids using electroporesis with a voltage of 1500 V at time constant of 5 ms [21]. 1 mL transformants were cultured on YPD plates containing 250 μg/ml G-418 at 30 °C for 1 day and then sub-cultured with 100-fold dilution. The overnight YPD subculture was transferred to 5 ml BMGY medium with initial OD_600_ = 0.2 and cultured overnight before transfer to BMMY inducible medium. The supernatant was collected for analysis after 2 days of induction at 30 °C [22].

For purification, cultures of AnP and mutant transformants were centrifuged at 12,000×g for 10 min to remove cell debris, and the crude phytases were concentrated using an Amicon centrifugal filter device (cutoff 10.000). The concentrates were purified using a DEAE-Sepharose column (3 × 15 cm) equilibrated with 10 mM sodium acetate buffer (pH 5.5). The proteins were eluted with elution buffer with a linear gradient of NaCl from 0 to 0.5 M. The fraction profiles of OD_280_ and phytase activity were confirmed to contain the desired protein peaks. The peak fractions were collected and stored at − 20 °C for further characterization [22]. Purified protein samples were subjected to SDS-PAGE, and enzyme concentration was determined by Bradford protein assay kit.

### Enzyme activity characterization

One phytase activity unit is defined as the amount of enzyme required to release 1 μmol phosphate from phytate per minute [23]. All assays in this work were performed in triplicate. Thermostability was assayed by measuring residual enzyme activity after incubation at 70, 80 and 90 °C for 1 h at a phytase-optimal pH 5.5.

Kinetic parameters (*K*_*m*_, *V*_*max*_, and *k*_*cat*_) for each phytase from a Michaelis-Menten rate expression were determined in sodium acetate buffer (200 mM, pH 5.5) at 37 °C. The reactions were monitored at 12 different concentrations of sodium phytate ranging from 0.05 to 2.5 mM [22]. Kinetic parameters were calculated by fitting to the Michaelis-Menten function using Origin 8.5.1.

### MD simulation details

The *Aspergillus niger* phytase X-ray crystal structure (PDB: 3K4P), which shares 97% sequence identity with the AnP phytase in the above material section, was used as the starting geometry for the AnP protein [24]. The SWISS-MODEL server was used to build the 18 point mutation AnP-M1 [25]. After a 1000-step energy minimization, all the systems were equilibrated for 5 ns in the NPT ensemble followed by another 5 ns equilibration in the NVT ensemble by restraining all heavy atoms. Afterwards, each system was simulated for 50 ns in the NPT ensemble (323 K/343 K, 1 bar). All systems were solvated with TIP3P waters in an octahedral box [26]. Sodium and chloride ions were added (100 mM) to neutralize the systems. Protonation states for histidines were determined by the UCSF Chimera program [27]. The GROMACS program suite version 4.5.7 and Amber ff99SB force field were used in all simulations [28, 29]. Bond length constraints were applied to all bonds that contained hydrogen atoms based on the LINCS protocol [30]. An integration step of 0.002 ps was used in all simulations. Electrostatic integrations were treated with Particle Mesh Ewald method with a cutoff of 0.9 nm with grid spacing for the FFT grid< 0.12 nm [31].

### Hydrogen bond analysis

Hydrogen bonds between mutational residues and nearby residues in all simulation systems were analyzed by using g_hbond in the GROMACS suite. Geometrical criterions which include donor-acceptor distance (≤0.35 nm) and hydrogen-donor-acceptor angle (≤30°) are used to calculate hydrogen bond. For each time frame, if both the donor-acceptor distance and the hydrogen-donor-acceptor angle satisfy the criterions, the number of hydrogen bond will be counted as 1, otherwise 0. The number of hydrogen bonds was calculated based on the whole 50 ns simulation (50,000 frames in total) in each system, and the error bar represents one standard error which was calculated based on the averaged number of hydrogen bonds every 10 ns in each system.

### Clustering analysis

Pair-wise clustering analysis was performed using gcluster in the GROMACS suite based on the root-mean-square deviation (RMSD) of the heavy atoms of protein. The snapshots to do the clustering analysis were selected 10 ps intervals from the simulation trajectories (5000 snapshots in each system).

### Calculation of mainchain deviations between AnP and AfP

Crystal structures of AnP and AfP were firstly superimposed according to the result of pair-wise sequence alignment. Mainchain deviations were calculated for all equivalent mainchain Cα atoms based on their orthogonal coordinates.

## Additional files

Additional file 1: **Figure S1.** Root-mean-square deviations (RMSD) of AnP and AnP-M1. RMSD of heavy atoms of AnP (black) and AnP-M1 (green) as a function of simulation time at 50 °C, and RMSD of heavy atoms of AnP (red) and AnP-M1 (blue) as a function of simulation time at 70 °C. (PDF 956 kb)

Additional file 2: **Table S1.** Oligonucleotide primers for AnP-M1 and AnP-M2. (DOCX 13 kb)

## Abbreviations

AfP: *Aspergillus fumigatus* phytase
AnP: *Aspergillus niger* PhyA phytase
*E. coli*: *Escherichia coli*
HAPs: Histidine acid phytases
MD: Molecular dynamics
