# Supplementary figures and images for: Enhancing thermal tolerance of Aspergillus niger PhyA phytase directed by structural comparison and computational simulation
Source: BMC Biotechnol. 2018 Jun 1;18:36. doi: 10.1186/s12896-018-0445-y (PMC5984770; doi:10.1186/s12896-018-0445-y)

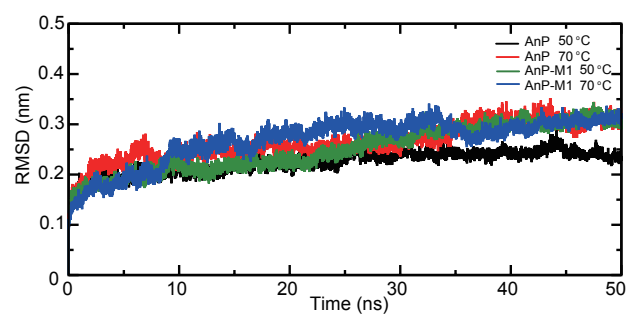

Supplement: Supplementary file 1 — Figure S1. Root-mean-square deviations (RMSD) of AnP and AnP-M1. RMSD of heavy atoms of AnP (black) and AnP-M1 (green) as a function of simulation time at 50 °C, and RMSD of heavy atoms of AnP (red) and AnP-M1 (blue) as a function of simulation time at 70 °C. (PDF 956 kb) [file 12896_2018_445_MOESM1_ESM.pdf]
