# Supplementary material for: Enhancing thermal tolerance of Aspergillus niger PhyA phytase directed by structural comparison and computational simulation
Source: BMC Biotechnol. 2018 Jun 1;18:36. doi: 10.1186/s12896-018-0445-y (PMC5984770; doi:10.1186/s12896-018-0445-y)
Supplement: Supplementary file 2 — Table S1. Oligonucleotide primers for AnP-M1 and AnP-M2. (DOCX 13 kb) [file 12896_2018_445_MOESM2_ESM.docx]

**Table S1 Oligonucleotide primers for AnP-M1 and AnP-M2.**

| Item | Primers |
| --- | --- |
| AnP-M1-1-1-forward | 5′- cgttcttctctctgg**a**a**g**acgaatcggtcatc-3′ |
| AnP-M1-1-1-reverse | 5′- **C**T**T**CCAGAGAGAAGAACGGTGCGTATT -3′ |
| AnP-M1-1-2-forward | 5′- tctctctggcaaacgaat**t**g**tc**c**g**tctcctctga -3′ |
| AnP-M1-1-2-reverse | 5′- **C**G**GA**C**A**ATTCGTtTgCCAGAGAGAAGAACGGTGC -3′ |
| AnP-M1-1-3-forward | 5′- cgaatcggtcatctcc**t**ctgaggtgc -3′ |
| AnP-M1-1-3- reverse | 5′- **A**GGAGATGACCGATTCGTTTGCCAGAGAG -3′ |
| AnP-M1-2-1-forward | 5′- Gctgaaggatcct**G**gtgcc**AC**gcccggccaat -3′ |
| AnP-M1-2-1- reverse | 5′- **GT**GGCAC**C**AGGATCCTTCAGCTTGGTGCTC -3′ |
| AnP-M1-2-2-forward | 5′- gctgaaggatcctcgtgcccag**AA**c**C**gcGCatcgtc -3′ |
| AnP-M1-2-2- reverse | 5′- **G**G**TT**CtgGGCACgAGGATCCTTCAGCTTGGTGCTC -3′ |
| AnP-M1-2-3-forward | 5′- tcctcgtgcccagcccggc**GC**atcgtcgccc -3′ |
| AnP-M1-2-3- reverse | 5′- **GC**GCCGGGCTGGGCACGAGGATCCTTCAGCAA -3′ |
| AnP-M1-3-1-forward | 5′- CTTCGacaccatctcca**GA**a**C**caccgtcgacac -3′ |
| AnP-M1-3-1- reverse | 5′- **G**T**TC**TGGAGATGGTGTCGAAGGAGCGAAGGAG -3′ |
| AnP-M1-3-2-forward | 5′- accatctccaccagca**G**cg**A**cg**C**caGcCagctgt -3′ |
| AnP-M1-3-2- reverse | 5′- **G**CG**T**CG**C**TGCTGGTGGAGATGGTGTCGGAGGAGC -3′ |
| AnP-M1-3-3-forward | 5′- ATCTCCACCAGCACCGTCGACA**G**c**C**agctgtcccc -3′ |
| AnP-M1-3-3- reverse | 5′- **G**G**C**TGTCGACGGTGCTGGTGGAGATGGTGTCGAAG -3′ |
| AnP-M2-1-1-forward | 5′- GGCAAACGAATCG**TC**CATCTCC**T**CTGAGGTGCCAGC -3′ |
| AnP-M2-1-1- reverse | 5′- **A**GGAGATG**GA**CGATTCGTTTGCCAGAGAGAAGAATG-3′ |
| AnP-M2-2-1-forward | 5′-TGAAGGATCCTCGTGCC**AC**GCCCGGCCAATCGTCGCC -3′ |
| AnP-M2-2-1- reverse | 5′- GGGC**GT**GGCACGAGGATCCTTCAGCTTGGTGCTCTG -3′ |
| AnP-M2-2-2-forward | 5′- AAGGATCCTCGTGCCCAG**AA**CGGCCAATCGTCGCCA -3′ |
| AnP-M2-2-2- reverse | 5′- CCG**TT**CTGGGCACGAGGATCCTTCAGCTTGGTGCT -3′ |
| AnP-M2-2-3-forward | 5′- ATCCTCGTGCCCAGCCC**A**G**A**CAATCGTCGCCAAAGAT -3′ |
| AnP-M2-2-3- reverse | 5′- ATTG**T**C**T**GGGCTGGGCACGAGGATCCTTCAGCTTGGT -3′ |
| AnP-M2-3-1-forward | 5′- CCTTCGACACCATCTCCA**GA**AGCACCGTCGACACCAA -3′ |
| AnP-M2-3-1- reverse | 5′-GCT**TC**TGGAGATGGTGTCGAAGGAGCACATGTCCATG -3′ |
| AnP-M2-3-2-forward | 5′- ATCTCCACCAGCACCG**A**CG**C**CACCAAGCTGTCCCCTT -3′ |
| AnP-M2-3-2- reverse | 5′- GGTG**G**CG**T**CGGTGCTGGTGGAGATGGTGTCGAAGGAG -3′ |
| AnP-M2-3-3-forward | 5′- CAGCACCGTCGACACC**C**AGCTGTCCCCTTTCTGTGAC -3′ |
| AnP-M2-3-3- reverse | 5′- GT**G**GCGTCGGTGCTGGTGGAGATGGTGTCGAAGGAGC -3′ |

Nucleotide resulting in the desired mutation is underlined and in bold.
